# Factors affecting demand for modern contraceptives among currently married reproductive age women in rural Kebeles of Nunu Kumba district, Oromia, Ethiopia

**DOI:** 10.1186/s40834-019-0103-3

**Published:** 2019-12-05

**Authors:** Alemu Kebede, Sileshi G. Abaya, Elias Merdassa, Tariku Tesfaye Bekuma

**Affiliations:** 1 Department of Public Health, Institute of Health Sciences, Wollega University, P. O. Box: 395, Nekemte, Ethiopia; 2 Public Health, Adama Hospital Medical College, Adama, Ethiopia

**Keywords:** Demand, Family planning use, Unmet need, Nunu Kumba, Married women

## Abstract

**Background:**

Family planning plays a key role in improving the health of the mother and the child. Yet there are still significant levels of demand for family planning that are unmet and these can lead to unintended pregnancy. So, women’s demand for contraceptive utilization to limit, space, or delay the number of family size can be increased by integrating family planning service at each service delivery points.

**Objective:**

The main aim of this study was to assess the demand for modern contraceptive and associated factors among currently married women of the reproductive age group in rural kebeles of Nunu Kumba District, 2015.

**Methods:**

A community-based cross-sectional study design was conducted from January 15–30, 2015 among 584 systematically selected currently married women of reproductive age in six rural kebeles of Nunu Kumba District. A pre-tested and interviewer-administered questionnaire was used to collect the data. Data were analyzed using SPSS version 20. Descriptive statistics were done to summarize the data. A multivariate logistic regressions analysis method was employed and odds ratio with 95% confidence interval was used to control for possible confounders. *P*-value < 0.05 was used to declare a significant association.

**Results:**

The total demand for modern methods of contraceptive was 450 (77.1%) of which 325 (55.7%) of them were current user and 125 (21.4%) of them were had unmet need for modern contraceptive methods. Being in the younger age group (15–24 and 25–34 years [AOR = 0.196; 95% CI: 0.055, 0.692] and [AOR = 0.179, 95% CI: 0.043, 0.745] respectively, husband having no intention for more children [AOR = 4.124, 95% CI: 1.891, 8.996], number of children alive [AOR = 2.617, 95% CI: 1.056, 6.486], and couples ever not discussed on family planning [AOR = 0.340, 95% CI: 0.187, 0.619] were factors associated with demand for modern methods of contraception.

**Conclusion:**

The total demand for modern methods of contraceptive was high in the study area except for long-acting and permanent methods with high unmet need for spacing than for limiting. Therefore, any program aimed at promoting family planning at the district level should look for ways and means of increasing demand for long-acting and permanent family planning methods and encouraging husband involvement to increase its utilization.

## Introduction

The need or demand for contraceptives, and meeting the demand or the supply of contraceptives are dependent on each other. As women’s demand for contraception increases, the need for governments, donors, manufacturers and other stakeholders to supply the demand becomes increasingly critical [1]. Improvements in meeting the demand for family planning require not only data on overall levels and trends in contraceptive prevalence and unmet need for family planning but also an assessment of the diversity of contraceptive methods used [2].

Researchers have shown that Reproductive age women have varying contraceptive needs. Because of side effects, daily intake and similar complain, many women do not use oral contraceptives effectively which can lead to unintended pregnancy. Rather, they choose long-term reversible contraception lik Intra Uterine Devices (IUDs) and implants [3]. The total demand for family planning is currently defined as the percent of married or in union women aged 15–49 years who want to delay or limit childbearing (i.e. the combination of women with unmet need and women using family planning constitutes the total demand for family planning) [4].

Yet there are still significant levels of demand for family planning that are unmet. If this unmet need were met, unintended pregnancies would be reduced, women’s health and lives would be improved, can prevent an estimated 2.7 million infant deaths globally and the consequent impact on fertility would result in lower population growth [5]. The growing use of contraception around the world has given couples the ability to choose the number and spacing of their children and has tremendous lifesaving benefits. But, contraceptive use is still low and the need for contraception high in some of the world poorest and most populous places [6]. Contraceptive use was increased worldwide over the last decade but, Africa has stilla high unmet need for family planning that approximately 25% of women and couples in Sub-Saharan Africa who wanted to space or limit their births are not using any type of contraception [7].

Half of the married women worldwide now use a modern method of contraception, but 200 million women still have an unmet need that they would like either to stop having children or delay their next birth for at least 2 years but are not using an effective contraceptive method. This unmet need is fueled by lack of information, fear of social disapproval or a husband’s opposition, religious or cultural beliefs, and concern for contraceptive side-effects or impacts on health. This could lead to increased unwanted pregnancy and induce abortion [8–10].

In many resource-poor settings, the growing unmet need for contraception is astounding. So, couples who wish to have fewer children are unable to determine the size of their families as funds for family planning continues to be scarce and existing programs and services fail to meet the concerns and desires of their users [11].

In Ethiopia, the strategy was emphasized on delivery of short-acting methods, especially pills where the probability of an adult woman dying from a maternal cause during her reproductive lifespan is about one in 40 [12, 13]. EDHS 2011 indicated that unmet need for contraceptives was about 25% with a total demand of 54% [14].

Although family planning use among women of reproductive age has been increased from its virtual nonexistence level, it is still low in Ethiopia. This is as a result of low availability of a variety of contraceptive methods [15]. Availability of quality family planning services, diversity of the methods and correct information enable women to make informed choices. This study has determined the level of family planning and identified factors associated with demand for family planning and hoped to be used as an input for program and health policymakers in designing family planning service delivery strategies in Ethiopia in general and in the study area specifically.

### Conceptual framework

**Fig. 1:**
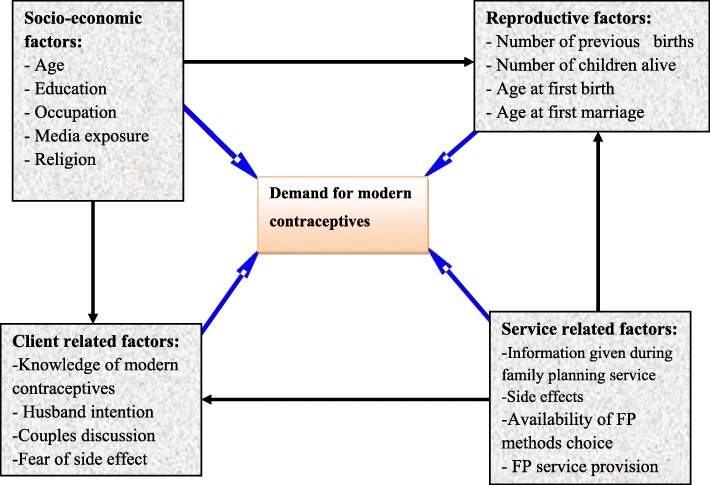
Conceptual Framework of demand for modern contraceptives of respondents in Nunu Kumba woreda, January 2015 (Adopted from Jane Bertrand framework, 2006).

The framework is conceptualized by using two groups of variables, the dependent and independent variables. The independent variables are those that influence demand for modern family planning (dependent variables) which include socio-economic, reproductive history and client-related factors (Fig. 1).

## Methods

### Study area and period

The study was conducted in Nunu Kumba District from January 15–30/ 2015. Nunu Kumba is 68kms away from Nekemte town, the capital of the zone, and 381kms from Addis Ababa, the capital city of Ethiopia. It is one of the 17 Districts in the East Wollega Zone. It is bordered on the Southwest by Jimma Arjo District, on the Northwest by Wayu Tuka District, on the Northeast by Wama River which separates it from Wama Hagalo District, and on the Southeast by Ilu Ababora zone. The administrative center of this District is Nunu. The data from District health office (2014) indicated that the district has 22 Kebeles; of which 2 were urban and 20 were rural and had an estimated total population of 78,541, of which 39,585 were males and 38,956 were females. In this District, there were 4 health centers and 20 rural health posts. Also, there were two secondary schools [9, 10] and one preparatory school (Nunu Kumba district Health office report, 2015).

### Study design

A community-based cross-sectional study design was employed using both quantitative and qualitative methods.

### Source population

All currently married women of the reproductive age group in rural kebeles of Nunu Kumba district were the source population.

### Study population

Randomly selected married women between 15 and 49 years of age who were living in selected rural kebeles of Nunu Kumba district.

### Inclusion and exclusion criteria

#### Inclusion criteria

- All married women of the reproductive age group in selected kebeles.
- Being a resident of selected kebeles at least for 6 months

#### Exclusion criteria

Married women of the reproductive age group who were critically ill, unable to talk or hear during the study period were excluded from the study.

### Sample size determination

#### Quantitative method

The sample size was determined using the formula for a single population proportion:

$$ n=\frac{{\left({Z}_{\alpha /2}\right)}^2\times \mathrm{pq}\times \mathrm{DE}}{{\mathrm{d}}^2} $$

Where: - α - the level of confidence

p - Percentage of total demand for family planning among currently married women of Butajira district, which is 77.8% [15]

q - (1-p);

n - Sample size.

Z - Standard normal distribution curve value for 95% CI which is 1.96 (where α = 0.05).

d - Tolerable margin of error = 5%

Hence

$$ \mathrm{n}=\frac{(1.96)^2\times 0.778\times 0.222}{{\left(0,05\right)}^2}\times 2=530.8 $$

DE - design effect of the study due to its multi-stage nature

Additional 10% allowance (non-response) for absenteeism and refusal to participate in the study was considered. Thus, 530.8 + 53.08 = 583.88; 584 married women were interviewed.

#### Qualitative part

Four sessions of focus group discussions were carried out among model married mothers and model husbands other than those who took part in the study. Individuals of similar backgrounds: age group, educational status, occupation, and residents of the study area for more than 6 months have been included in the same group. After participants were identified, appropriate time and comfortable place of meeting were selected and organized. All the discussions were moderated by a principal investigator with one trained recorder and one note taker health professionals (BSc). A The semi-structured, open-ended questionnaire was used to initiate discussion and all the discussions were undertaken in *Afan Oromo* which is the local language of discussants.

### Sampling procedure

#### Quantitative study

**Fig. 2:**
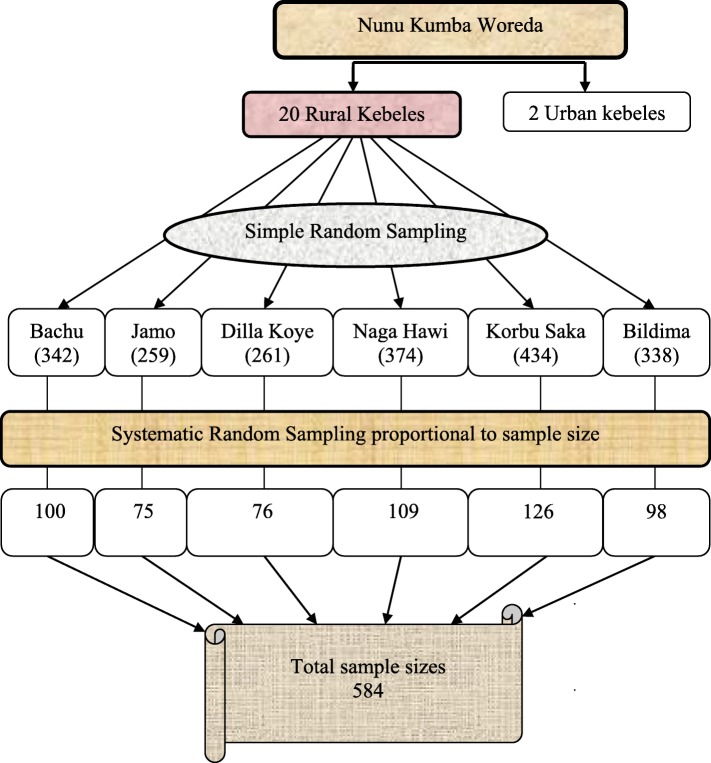
Schematic presentation of sample size (for quantitative study) selection procedure of Nunu Kumba Woreda, January 2015

Firstly, 20 rural kebeles were listed and using simple random sampling technique, 6 rural kebeles were selected. Then, the sample for the respective selected kebeles was allocated proportionally to their population size of mothers in reproductive age groups. Using a sampling frame fromthe family folder in the health posts and any updated information that was added from HEWs in the selected kebeles, actual sample for each selected kebele was determined. Accordingly, 584 currently married women of reproductive age group were selected from all selected kebeles using systematic random sampling technique. In case, when the household in which eligible mother resides was closed during data collection or the eligible mother was absent on the date of data collection, interviewers were revisited that household three times at different time intervals and interviewed (included) these mothers (Fig. 2).

#### Data collection tools and data collection procedures

##### Data collection tools

Data was collected by interviewing married women of reproductive age group face to face using structured, standardized and interviewer-administered questionnaire adapted from EDHS and modified based on the objective of the study. The questionnaire contains questions addressing the demand for modern contraceptive methods and factors associated with contraceptives need among married women of the reproductive age group in the study area. It was prepared in English and translated to Afan Oromo, and back-translated to English by different experts (high school Language teachers) to ensure consistency. In addition, FGD guide was prepared in English and translated to Afan Oromo was used directly as a guide for the FGD.

##### Data collectors

Data was collected by six female grade 12 completed students and supervised by three health professionals (degree holders). Both the interviewers and supervisors were given two-day training before the actual work about the aim of study which was assessed the level of modern contraceptives demand and associated factors that were helped for the amendment of the identified gap with the concerned body; and data collection techniques by going through the questionnaires question by question.

##### Operational definitions

- **Total demand for family planning:** percent of married or in union women aged 15–49 years who want to delay or limit childbearing
- **Long-acting and permanent methods of contraception (LAPMs):** those methods that prevent pregnancy for three and more years per application (Implants, IUCD, male and female sterilizations).
- **Demand for modern contraceptives:** current use of modern contraception plus the unmet need for modern contraception
- **Unmet need for family planning:** women who want to avoid a pregnancy but are not using an effective method of family planning
- **Postpartum amenorrhoeic:** Women whose menstrual period has not returned since their most recent birth, and who gave birth in the last 2 years (0–23 months).
- **Infecund:** Women, who had first married five or more years ago, never used contraception, and have not had a birth in the past 5 years.

### Variables

#### Dependent variables

- Demand for modern contraceptives

#### Independent variables

- Socio-demographic (economic) characteristics - such as the age of woman, religion, Educational status of woman and husband, ethnicity, occupation, Parity
- Knowledge about modern contraceptives
- Husband/partner influence
- Choice of method
- Number of children alive
- Information is given to clients during family planning service
- Contraceptives side effects

#### Data processing and analysis

After data collection, each questionnaire was checked by the supervisors. Then, the actual data was entered and analyzed by SPSS version 20 statistical packages. To check missing values and outlier’s frequency output was used and cleaning was done. Descriptive statistics were done to summarize the data. A bivariate analysis was used to identify candidate variables and a multivariate logistic regression model was used to control for possible confounding variables and predict population parameters. Crude and Adjusted Odds ratio with 95% confidence interval was used to assess the association of dependent and independent variables. All the assumptions of the models (normality of the data and multi-collinearity) were checked to be satisfied. Regarding focus group discussions, the recorded audio was transcribed verbatim and the note was reviewed. Thematic content analysis was used in that texts were categorized into primary themes. Then it was compiled into broader concepts. Besides, quotes of participants that exemplify key concepts were used directly during analysis. Finally, the concepts were developed into major themes under each discussion guides.

#### Data quality management and control

The quality of data was assured by properly designing and pre-testing of the questionnaire; proper training of the interviewers and supervisors on data collection procedures: proper categorization and coding of the questionnaire. The questionnaire was reviewed every day and checked for completeness by the immediate supervisors and principal investigator, and the necessary feedback was given to data collectors in the next morning before data collection.

#### Pre-testing

The questionnaire was pre-tested in one of the kebeles other than the selected study area (in Jima Arjo District, Wayu Abayi kebele), but has similar socio-demographic characteristics with the study population. Accordingly, it was carried out among 5% of the total sample size and necessary modifications were made to the questionnaire before actual data collection.

#### Ethical consideration

Ethical clearance was obtained from the Research Ethics Committee of Wollega University. Then, support letter was written from the District Health Office and given to each Kebele administrators and verbal consent was taken from each eligible woman. Study subjects were informed that the study was not had any risks. In addition, the objective and benefits of the study were explained to them. Identifying information (like name, phone number, etc) was excluded from the questionnaire to ensure privacy and confidentiality. The right of individual not to participate in the study was also be respected.

## Results

### Quantitative study

#### Socio-demographic characteristics of respondents

A total of 584 currently married women in the reproductive age were responded for the study making the response rate of 100%. Majority of the respondents were between the ages of 35–39 years. The mean age of respondents was 31.26 years with + 6.53 years standard deviations. Majority of the study subjects were Oromo in ethnicity 570 (97.6%) and 329 (56.3%) of them were Protestant followers. Concerning educational status, 29 (5%) of respondents were secondary school and above, and 367 (62.8%) of them were illiterate. On the other hand, majority 264 (45.2%) of respondents’ husband were unable to read and write; and 108 (18.5%) of them were grade 5–8. Two hundred ninety-seven (50.9%) of respondents werehousewives and four of them were a governmental employee. About 50% of the respondents earned less than 1300 and 25% of them earned less than 950.00 ETB (Table 1).

**Table 1:** Socio-demographic characteristics of respondents in Nunu Kumba District, January 2015

Variables (*n* = 584)	Frequency	Percent
Age		
15–19	13	2.2
20–24	77	13.2
25–29	141	24.1
30–34	144	24.7
35–39	146	25.0
40+	63	10.8
Ethnicity		
Oromo	570	97.6
Amhara	12	2.1
Gurage	2	0.3
Religion		
Orthodox	211	36.1
Muslim	44	7.5
Protestant	329	56.4
Respondents educational status		
Unable to write and read	367	62.8
Read and write	29	5.0
Grade 1–8	159	27.2
Grade 9–12	25	4.3
College and above	4	0.7
Respondents occupational status		
House wife	297	50.9
Student	23	3.9
Farmer	260	44.5
Government employee	4	0.7
Family monthly income in quartile	Birr (ETB)	Frequency
First quartile	950	146
Second quartile	1300	292
Third quartile	1850	438

#### Reproductive history of the respondents

Out of the total respondents, 268 (45.9%) were married between the age of 15–19 years and 298 (51%) of the respondents were married between the age of 20–24 years old. Five hundred twenty-three (89.6%) of the respondents had ever been pregnant, out of which 391 (74.9%) of them were between 20 and 24 years of age at first birth. Three hundred seven (52.6%) respondents decided the total number of children they would like to have in their lifetime with TFR 5.1, out of which 120 (39.1%) and 186 (60.6%) of them would like to have 3–4 and above 4 children in lifetime respectively (Table 2).

**Table 2:** Reproductive history of the respondents in Nunu Kumba district, January 2015

Variables (*n* = 584)	Frequency	Percent
Age at first marriage		
15–19	268	45.9
20–24	298	51.0
25–29	17	2.9
30–34	1	0.2
Age of mother at first birth (*n* = 523)		
15–19	72	13.8
20–24	391	74.7
25–29	59	11.3
30–34	1	.2
Number of live births (*n* = 522)		
< 5	412	78.9
> =5	110	21.1
Number of children alive (*n* = 521)		
1–2	145	27.8
3–4	188	36.1
5+	188	36.1
Number of children respondents would like to have in a lifetime (*n* = 307)		
2	1	0.3
3–4	120	39.1
> 4	186	60.6

**Fig. 3:**
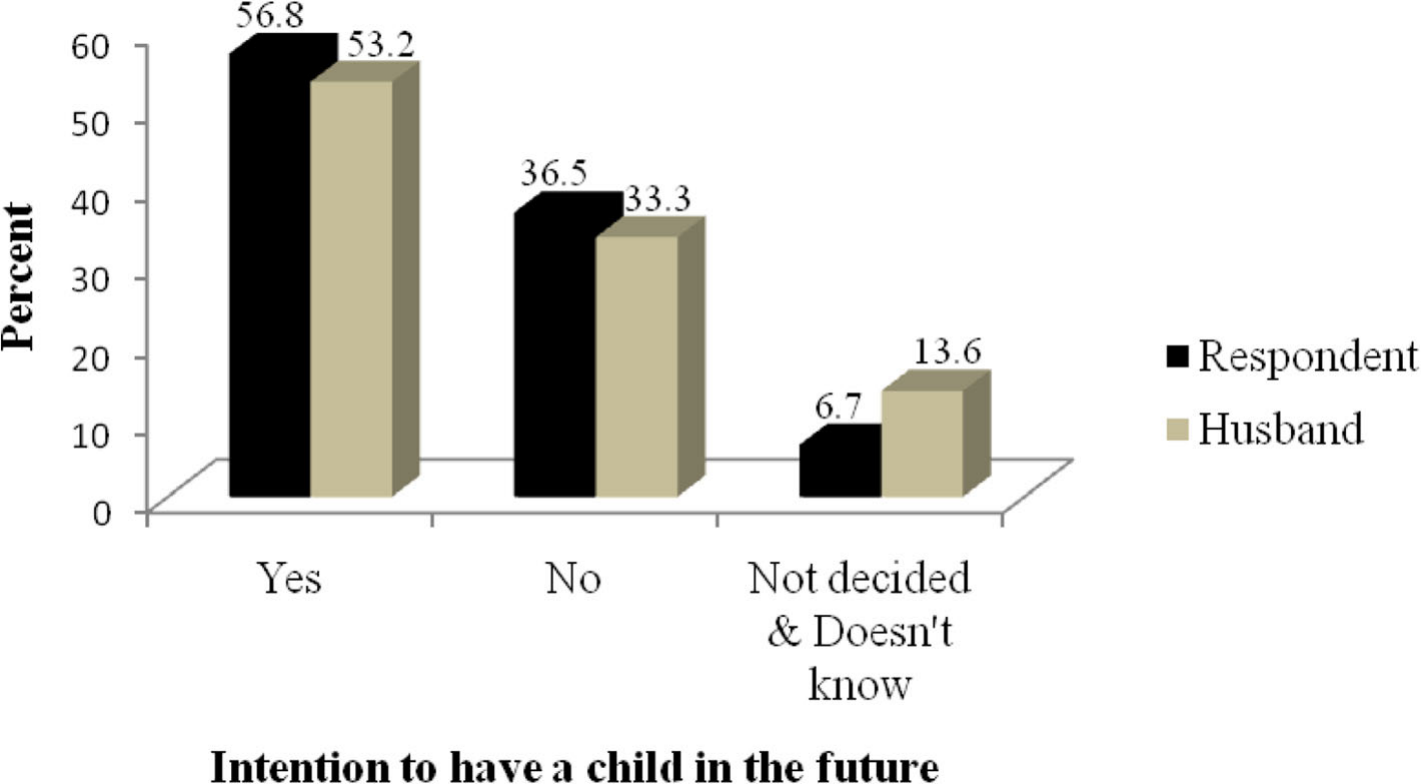
Respondents’ and their husbands’ intention (as reported by the respondent) to have a child in the future in Nunu Kumba woreda, January 2015

Five hundred twenty-three (89.6%) women have given one or more births of which only two women have no live child duringthe data collection period. In addition, it was mentioned that 79 (15.1%) have a history of stillbirths and 51 (9.8%) have a history of abortion. Out of 523 nonpregnant women, 297 (56.8%) intended to have children in the future, 191 (36.5%) mentioned that they did not need children and 35 (6.7%) have not decided their future intention to have children. Similarly, 278 (53.2%) of them knew their husband intention while 71 (13.6) did not know (Fig. 3).

Among those who intended to have children in the future, 185 (62.3%) were because of few children they have, 73 (24.6%) of them wanted more sons and 33 (11.1%) wanted more daughters, while six (2%) of them were because of children died.

#### Knowledge of women about modern contraceptive and source of information

Five hundred seventy-three (98.1%) of the respondents have heard at least one method of modern family planning. Health workers 573 (100%) and radio 201 (35.1%) were the most commonly mentioned source of information respectively. Public health facilities (99.5%) were the most common place where the information about family planning method can be gained. Regarding the advantage of family planning methods, 457 (79.9%), 386 (67.5), 92 (16.1%) knew/mentioned to limit family size, to protect unwanted pregnancy and to prevent STIs, respectively. The most commonly known family planning methods were injectables (96.2%), followed by pills (94.4%), implants (88%) and condom (22.2%). Five hundred nineteen (90.7%) respondents mentioned that modern contraceptive methods are the best method to prevent unwanted and mistimed pregnancy. Two hundred sixty-seven (46.6%) respondents had discussed about family planning with their husband in the last 12 months. Out of the total respondents, 206 (35.3%) discussed about family planning with health care providers and visited health facility in the last 12 months for different family planning related services (Table 3).

**Table 3:** Knowledge/awareness of respondents on family planning in Nunu Kumba district, January 2015

Variables	Frequency	Percent
Heard about FP (*n* = 584)		
Yes	573	98.1
No	11	1.9
Source of contraceptives mentioned by respondents		
Public health facility	570	99.5
Private health facility	99	17.3
NGO health facilities	12	2.1
Contraceptives methods are known by respondents		
Pills	541	94.4
Injectables	551	96.2
Implants	504	88.0
IUCD	292	51.0
Permanent FP	134	23.4
Condom	127	22.2
Couples discussion about family planning within last 12 months (*n* = 573)		
Yes	267	46.6
No	306	53.4

NB: Percentage may not add up 100% as multiple responses are possible for the variables of sources of contraceptives mentioned and contraceptive methods known by respondents

#### Attitude towards family planning utilization

Women were asked to reflect their opinion on certain attitude questions. Five hundred sixty (95.9) of them agreed that if the family uses contraceptives, children will have better opportunities for education, while 2.4% of them disagreed on the idea. Similarly, out of total respondents, 95.5% of them believed that family planning helps to improve one’s standard of living and 11 (1.9%) of them were neither agreed nor disagreed. In the same way, 93.8 and 94.7% of the respondents agreed that family planning helps the mother to regain strength before her next birth of baby and child spacing helps protect the health of children and mothers, respectively. Sixty-nine (11.8%) of the respondents agreed that family planning causes loss of confidence between husband and wife. Fifty (8.6%) of the respondents agreed that husbands will abandon wives who practice family planning. More than three-fourths (458) of them believed that using contraceptive may cause infertility for women. Five hundred forty-seven (93.7%) agreed that a couple who practices family planning has a happy family. Out of the study participants, 71.6% of them discussed family planning issues with their husband and 82.9% of them made a decision about family planning with their husbands. Moreover, 22.1% of them agreed in having many children are advantageous (Table 4).

**Table 4:** Attitude of respondents towards family planning in Nunu Kumba district, January 2015

Variables (*n* = 584)	Agree No. (%)	Disagree No. (%)	Neutral No. (%)
If family use contraceptive, children will have better opportunities for education	560 (95.9)	14 (2.4)	10(1.7)
FP helps to improve one’s standard of living	558 (95.5)	15 (2.6)	11 (1.9)
FP helps the mother to regain strength before her next birth of a baby	548 (93.8)	28 (4.8)	8 (1.4)
Child spacing helps protect the health of children and mothers	553 (94.7)	26 (4.4)	5 (0.9)
FP causes loss of confidence between husband and wife	69 (11.8)	496 (84.9)	19 (3.3)
Husbands will abandon wives who practice FP	50 (8.6)	509 (87.2)	25 (4.3)
Contraceptive use may cause infertility in women	104 (17.8)	458 (78.4)	22 (3.8)
A couple who practices FP has a happy family	547 (93.6)	32 (5.5)	5 (0.9)

#### Contraceptive utilization among currently married women

**Fig. 4:**
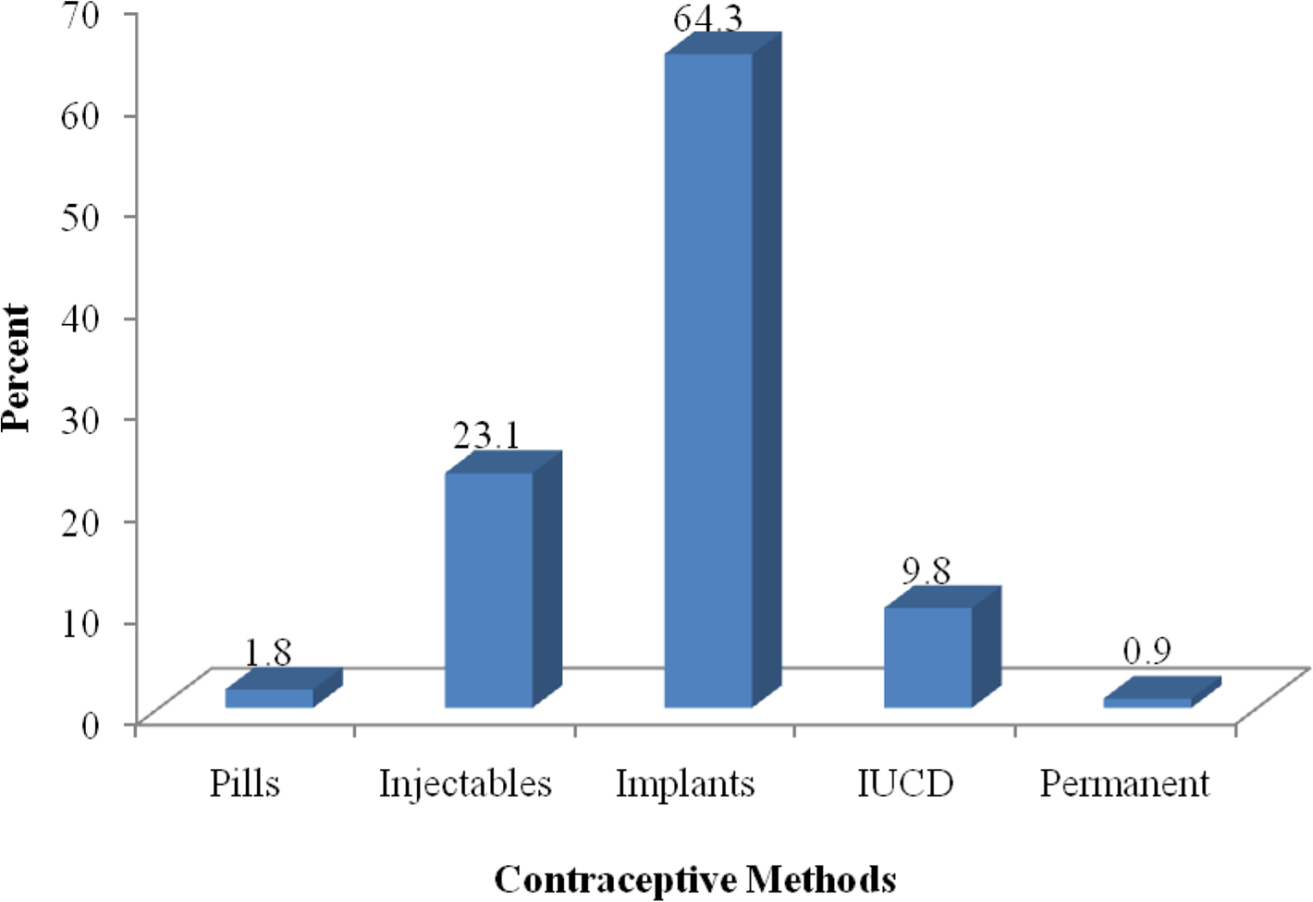
Distribution of modern contraceptive methods being used by respondents in Nunu Kumba woreda, January 2015

From all respondents, 325 (55.7%) have been using modern contraceptive methods. The majority, 209 (64.3%) were using Implant followed by Injectable 75 (23.1%) and only three respondents were using permanent method (Fig. 4).

**Fig. 5:**
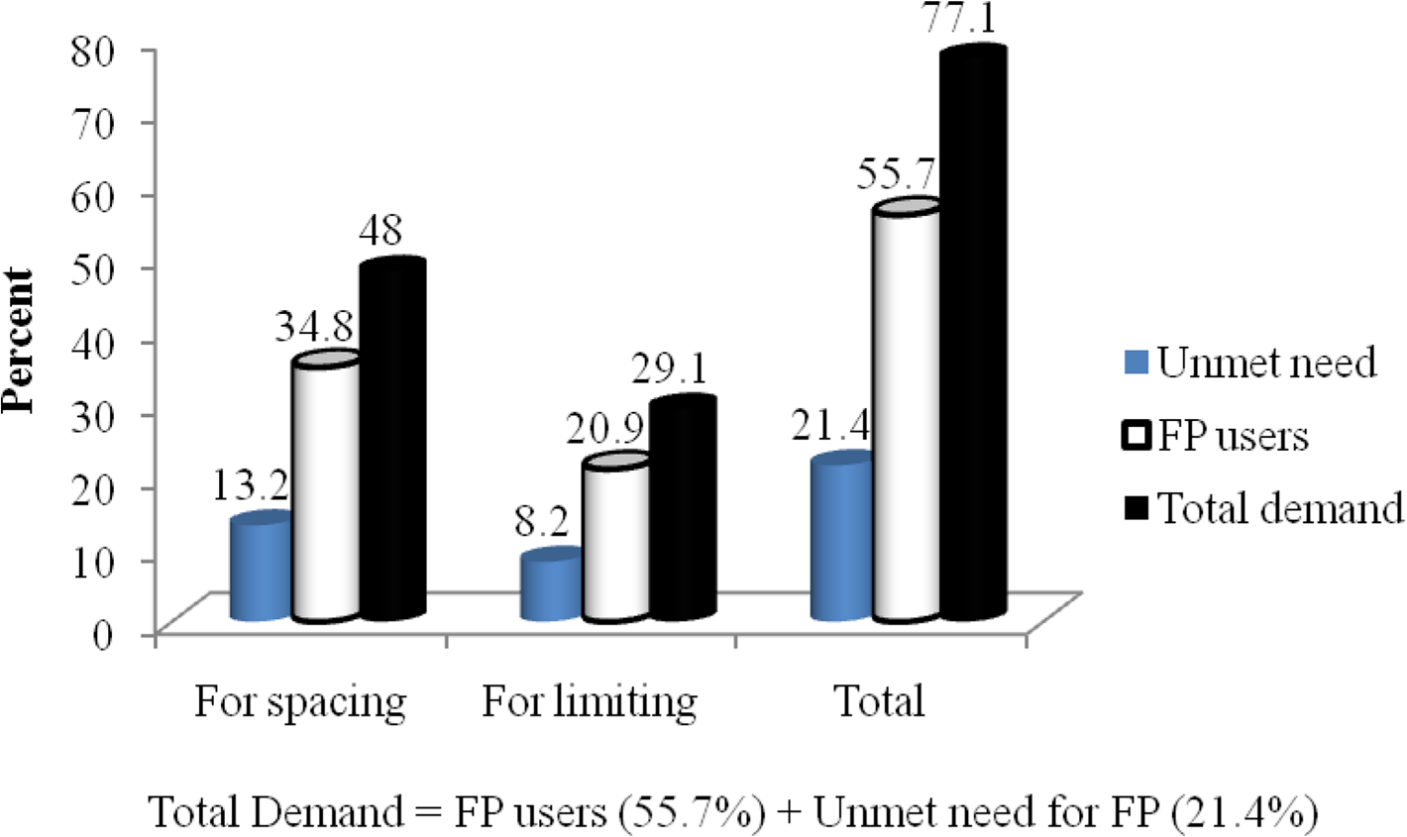
Total demand for modern contraceptives among respondents in Nunu Kumba woreda, January 2015

Unmet need for family planning was found to be 125 (21.4%) out which 77 (13.2%) was for spacing and 48 (8.2%) was for limiting (Fig. 5).

**Fig. 6:**
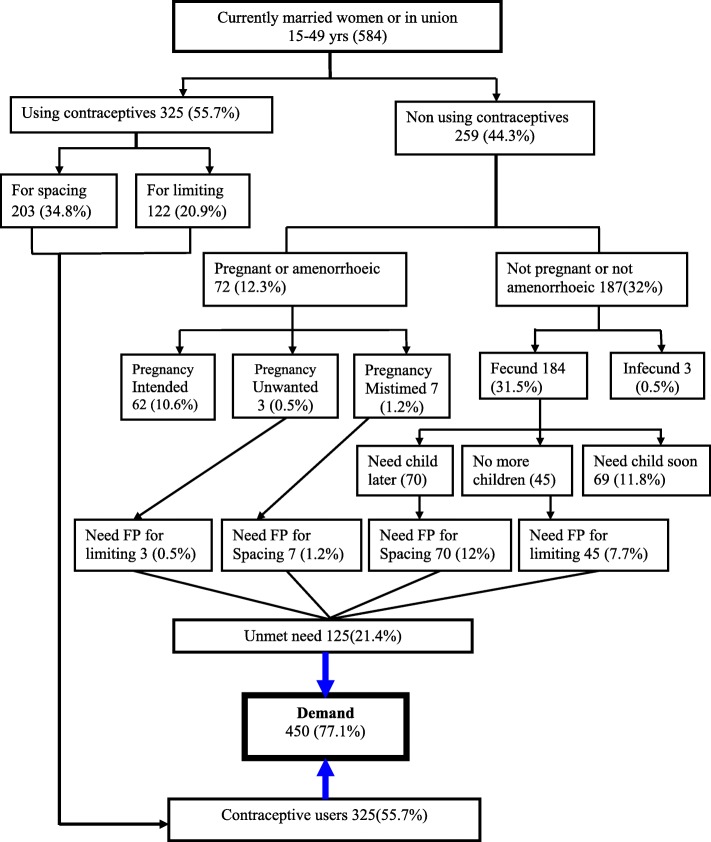
Model for determination of demand for the modern contraceptive in Nunu Kumba woreda, January 2015 (Bradley and F. West off et al. 2012)

In this study, the total demand for modern contraceptives were 450 (77.1%), out of which 325 (55.7%) were family planning users, while 125 (21.4%) had unmet need for family planning. Out of the total family planning users, 203 (34.8%) were using contraceptives for spacing and 122 (20.9%) were using for limiting (Fig. 6).

Concerning current pregnancy status of the respondents, 61 (10.4%) were pregnant out of which 51 (83.6%) were intended and 7 (11.5%) were mistimed while the three (4.9%) were unwanted.

About eight out of ten respondents with unintended pregnancies stated that they became pregnant due to fear of contraceptive side effects. But, all respondents in postpartum amenorrheic mentioned that their last pregnancy was intended. From the total, 187 of non pregnant, non family planning user and not postpartum amenorrheic women, 184 (98.4%) replied that they were fecund and regarding their future intention to have a child, it was mentioned that 70 (37.5%) of them want later, 69 (36.8%) of them want soon and 45 (24.5%) of them want no more children. Three respondents have considered themselves as infecund.

Out of 256 non- family planning users, 160 (62.5%) of them have intended to use a contraceptive, 79 (30.9%) of them have not intended to use and the rest seventeen (6.6%) of them have not decided their future intention to use modern methods of contraceptives. Regarding intention on family planning utilization, out of those who have not intended to use family planning, 51 (64.6%) of them were as a result of fear of side effects and only one respondent was as a result of husband refusal (Fig. 6).

Concerning demand for the modern contraceptives, this result indicated that from the total mothers interviewed, demand for the modern contraceptives was 450 (77.1%) out of which 325 (55.7%) was met that they were using contraceptives while the rest 125 (21.4%) was unmet since they were in need either to limit or space child births (Fig. 6).

#### Factors associated with demand for modern contraceptive methods

Bivariate and multivariate logistic regression was used to identify variables associated with the demand for modern contraceptives. Accordingly, in the bivariate/unadjusted analysis, demand for modern family planning was associated with many variables like age, educational status, presence of communication materials in the house, estimated monthly income, number of children ever born, number of children alive, spousal discussion in the past 12 months about contraception, spousal ever discussed about contraception, and husband intention to have children. But, age at first marriage, number of pregnancy, age at first birth, number of living male female children, educational status of husband and decision maker on in the family on family planning were variables not associated with demand for modern methods of contraceptive (Table 5).

**Table 5:** Bivariate analysis of factors not associated with Demand for modern methods of contraceptives among respondents in Nunu Kumba district, January 2015

Variables	Response	Demand for FP		Crude OR (95% C.I.)	*P*-value
		Yes	No		
Number of pregnancy	< 5	308	72	1.000	0.000
	> = 5	116	27	1.004 (0.625, 1.641)	0.986
Age at first birth	15–24	48	12	1.000	0.000
	25–34	376	87	0.926 (0.472, 1.816)	0.822
Number of living female children	< 5	5	2	1.000	0.000
	> = 5	346	74	0.535 (0.102, 2.09)	0.459
Number of living male children	< 5	3	1	1.000	0.000
	> = 5	386	76	0.591 (0.061, 5.755)	0.650
Age at first marriage	< 18	276	92	1.000	0.000
	> = 18	174	42	0.724 (0.480, 1.093)	0.124
Husband educational status	Can’t read and write	147	48	1.000	0.899
	Read and write	62	18	1.009 (0.554, 1.837)	0.977
	Elementary	205	59	1.161 (0.472, 2.855)	0.745
	High school and above	36	9	0.889 (0.479, 1.649)	0.709
Decision maker on FP use	Woman	36	11	1.000	0.001
	Husband	414	123	1.028 (0.508, 2.081)	0.938

*OR* Odds ratio

After adjusting for confounding by multivariate analysis, factors that remained significantly associated with demand for modern contraceptive methods were: age of respondents, number of children alive, husband intention to have children, spousal discussion in the past 12 months about modern contraceptive and respondent ever discussed about contraception with their husband.

Women whose age 15–24 years were 0.8 times less likely to have demand for modern contraception than women whose age were in between 35 and 49 years [AOR = 0.196, 95%CI: 0.055, 0.692]. Similarly, women whose ages were between 25 and 34 years were almost 0.8 times less likely to have demand for modern contraception than women whose ages were in between 35 and 49 years [AOR = 0.179, 95%CI: 0.043, 0.745]. This showed that age and demand for modern contraceptive methods have a positive association. This study revealed that women who had 2–3 children were almost three times more likely to have demand for modern methods of contraceptive than women who had no and one child. [AOR = 2.617, 95%CI: 1.056, 6.486].

Regarding future intention, women whose husband do not want more children were about four times more likely to have a demand for modern contraception than those who intended to have more children [AOR = 4.124; 95%CI: 1.891, 8.996]. Similarly, women who did not know their husbands’ future intention to have children were more than four times more likely to have demand for modern methods of contraceptive than those who desired more children [AOR = 4.403; 95%CI: 1.763, 10.995]. This result showed a negative association between future intention for more children and demand for family planning.

There was a positive association betweenthe demand for modern contraception and spousal discussion about family planning. However, the spouse who had not discussed about contraception in the past 12 months haswas 0.5 times less likely to have demand for modern contraception than those who had discussed about family planning [AOR = 0.472; 95%CI: 0.255, 0.874]. Furthermore, there was an association between the demand for modern methods of contraception and couples ever had discussed about contraception. Women whoever had not discussed about family planning with their husband were 0.7 less likely to have demand for modern methods of contraception than those who ever had discussed [AOR = 0.340; 95%CI: 0.187, 0.619] (Table 6).

**Table 6:** Multivariate analysis of factors associated with demand for modern contraceptives among respondents in Nunu Kumba district, January 2015

Variables	Demand for FP		Crude OR (95% C.I.)	Adjusted OR (95% C.I.)
	Yes	No		
Age of women				
15–24	55	35	0.295 (0.167, 0.521)	0.196 (0.055, 0.692)*
25–34	219	66	0.622 (0.392, 0.988)	0.179 (0.043, 0.745)*
35–49	176	33	1.000	1.000
Women educational status				
Can’t read and write	314	82	1.000	1.000
Elementary	112	47	0.622 (0.410, 0.946)	0.939 (0.515, 1.712)
High school and above	24	5	1.254 (0.464, 3.386)	1.416 (0.255, 7.850)
Exposure to media				
Yes	272	65	1.000	1.000
No	178	69	0.616 (0.418, 0.909)	0.630 (0.373, 1.064)
Number of children alive				
< =1	49	22	1.000	1.000
2–3	132	31	1.912 (1.011, 3.616)	2.617 (1.056, 6.486)*
> =4	243	44	2.480 (1.365, 4.503)	2.444 (0.707, 8.456)
Husband intention to have children				
Yes	205	73	1.000	1.000
No	156	18	3.086 (1.769, 5.383)	4.124 (1.891, 8.996)**
Don’t know	63	8	2.804 (1.282, 6.134)	4.403 (1.763, 10.995)**
Couples ever discussion about FP				
Yes	352	66	1.000	1.000
No	98	68	0.270(0.180, 0.406)	0.340 (0.187, 0.619)**
Couples discussed about FP in last 12 months				
Yes	238	29	1.000	1.000
No	207	99	0.255 (0.162, 0.401)	0.472 (0.255, 0.874)*

*NB: 1.000* Reference category, *OR* Odds ratio, *CI* Confidence Interval

*P-value < 0.05; **P-value < 0.01

### Qualitative study results

A total of 33 discussants participated in four FGDs; two with men and two with women groups; each FGD containing 8 discussants on average. The FGDs were conducted separately for community members (women of reproductive age, husbands/men). The purpose and the content of FGDs were explained first and participants participated voluntarily. Nineteen of the participants were in the age group of 25–29 years and almost all were Protestants and seventeen of the participants were housewives by occupation.

In the group discussions, it was found that the majority of participants in the study had awareness about modern methods of contraception. They also recognized that the modern method of the contraceptive can protect unintended pregnancy and help to determine the number of children they want. They mentioned as they prefer to have 3–4 children in their lifetime. Some of the discussants using contraceptives were those having more than four children and they considered that having more than four children is not good.

Most of the discussants mentioned that older women are using family planning than younger women who want to have a child soon. In relation to this, a 32 years old, Para four women said that, “I knew many women in neighbor with older age and who have four and above children are using contraceptive methods to space or limit their family size, but women who have few children or no son or daughter are not using family planning because they need additional child.” (Literate, Implant user).

Furthermore, some of the discussants also mentioned that Injectables are safer than other methods as it has minor side effects like gastritis, menstrual cycle disturbance, and dizziness; and some of them had limited knowledge of permanent methods; and relied highly on negative and intangible ideas from friends and neighbors. There was a strongly expressed fear of procedures, misunderstanding of procedures, and side effects like headaches, bleeding changes, and weight gain for modern methods of contraception, especially, Implants and Injectables.

One discussant said, “I did not have any understanding of male and female sterilization; how is that? The implant is very dangerous, I myself saw it on my friend, and I feared it very much. It moves in the body even to the head. Sterilization is alsoharmful, I want to do that, but I fear it because I heard that heavy bleeding can happen during the procedure and it may cause uterine problems. I want to stop birth, but it is said that it is not good for health and you should have an adequate diet. How would those poor people, who couldn’t eat well, use it?” (A 35 years’ women, grade 2, Para 7, Injectable user).

Moreover, both women and men discussants said, “Couples should support each other and contraceptive use should be decided together by the couples.” But, they were still concerned about the side effects, return of fertility after taking implants and IUCD, insertion and removal procedures and effect on physical activities as well. They also associate it with the type of food they were taking. In addition, they had a fear of resistance to ingimplants by the family planning service providers. Some women had concerns such as need for a vaginal examination, discomfort during sex, fear of privacy, side effects (infection), and effects on long-term fertility if used IUCD.

For example, a woman discussant said, “After using IUCD, you may not give birth even it is said that it can stop birth. IUCD can result in sterility and can move into the abdomen. The implant also has pain and may be difficult to remove if it is covered by muscle. I saw my friend suffered from this problem. The implant can make the injected hand numb and you can’t rise and carry heavy things by that hand; how can they use these methods?” (A 33 years old, illiterate, Para 5, Injectable user).

In general, the discussants pointed out for the public to be interested in using modern contraception methods, especially long-acting and permanent methods in the future. They also indicated that further information on family planning service should be disseminated to the community by health professionals for the purpose of continuous awareness creation and attitudinal change in the public towards family planning utilization and misconceptions.

## Discussions

This study has attempted to assess demand for modern methods of contraceptive and associated factors among married women of reproductive age in Nunu Kumba district, rural kebeles. A total of 584 participants have been involved in the interview and 33 discussants participated in FGDs.

This study revealed that 77.1% of respondents had demand for modern contraceptive methods; of which 21.4% had an unmet need for modern contraceptives (13.2% for spacing and 8.2% for limiting) and 55.7% were currently using modern methods (34.8% for spacing and 20.9% for limiting). This finding is lower than the study conducted at rural part of Enemay district, Northwest Ethiopia reported 81.5%, but higher than the findings of the study conducted at Misha district, Southern Ethiopia reported 58.3% [16, 17]. These variations might be attributed to the expanding health services coverage and increased awareness of family planning and maternal health services.

Regarding demand for family planning, this finding is slightly lower than study done in Rwanda [13] (79%) of which 33% for spacing, 46% for limiting, but higher than that of study in Tanzania (2010) reported 60% of which 34% were using contraception methods and with unmet need of 26% [6]. This might be as a result of the time difference, accessibility, and availability of the family planning service provisions.

On the other hand, demand for family planning in this finding was similar to study conducted in Butajira which was 77.8% of which 25.4% were current users and 52.4% were unmet need for family planning, but greater than that of EDHS 2011 (50.9%) of rural married women respectively [14, 15]. Additionally, demand for modern contraception in this study was higher than the study done in Southern Ethiopia, Misha district (57.7%), of which 31.2% was contraceptive users (to space or limit their birth) and 26.5% had an unmet need for family planning [16]. This might be due to the difference in geographical and accessibility of a variety of family planning methods and the difference in time of investigation. Medias are also promoting modern contraception which can increase the acceptance and utilization of modern contraceptive methods.

Regarding the age of the respondents, this result indicated that women whose age were between 15 and 24 years and 25–34 years were less likely to have demand for modern contraception than women whose age were in between 35 and 49 years. Similar results from focus group discussion indicated that women of older age and multi Para have more demand for contraceptives in comparison with younger women who have few or no children. This showed that women age is positively associated with contraceptive prevalence. The reason for the age difference could be due to those women who were older were having more children and have more desire to limit or space the number of pregnancy than younger who had none or few children. This result is supported by studies done in Debre Markos and Goba, Bale Zone in which women who want more children were younger [3, 18]. Furthermore, a study done in most Sub-Sahara African countries showed that demand for family planning and patterns for unmet need by parity were similar to those by age because, as would be expected, age and parity are closely linked. This implies that unmet need for spacing decreases with age while unmet need for limiting increases, with slightly lower levels of unmet need among the oldest group of women, who have reached menopause, at which point they no longer need family planning at all [19].

Regarding future fertility intention, women whose husbands did not want more children were four times more likely to have demand for modern contraception than those who desire more children. This implies that those who have many children do not want more children and prefer to limit their number of children rather than spacing. So, as the number of children increases, the demand for modern methods of contraceptives utilization also increases. This result is consistent with the findings from a study done in Debre Markos [18].

This study also showed that women who had 2–3 children were almost three times more likely to have demand for modern methods of contraceptive than women who had no or one child. The reason for deference might be due to the more child the woman is having, the more likely she wants to space or limit the number of child and the more she was using contraceptive methods or had an unmet need. This study is in line with the study conducted in Debre Markos, north west Ethiopia which showed that women who have three and above children had more demand for family planning than those having less than three children [18]. Similarly, results from focus group discussion showed that women who had more than four children were using family planning to limit and space their children.

Regarding a couple discussions, 71.6% of them discussed family planning issues with their husband and 82.9% of them made a decision about family planning with their husbands. This study indicated that there was a positive association between the demand for contraception and spousal discussion about contraception. This result is supported witha study conducted in Misha district Southern Ethiopia in which discussion between couples on fertility issues was strongly associated with the use of contraceptives, indicating the importance of frequent discussions [16].

With regards to factors associated with the demand for modern contraceptives, the result from FGDs showed that couples discussion about family planning utilization is very important. Women who make the discussion about family planning with their husbands had more demand or lesser unmet need for contraception. This is due to the fact that husband-wife communications on family planning provide an enabling environment for women to implement their fertility desires and contraceptive needs.

Furthermore, women who had ever discussed family planning with their husband were more likely to discuss on the desired family size they want to have and know their husband’s perception towards modern contraceptive methods than those who had no discussion with having unmet need for the methods. The result is in line with studies done in Goba, Debre Birhan District (North Shoa Zone) and Tanzania in which contraceptive practice was found to be associated with a spousal discussion about family planning and women’s perception of husband’s approval of contraceptive practices [3, 6, 18].

### Strength

Using both quantitative and qualitative methods is the strength of the study.

### Limitation

Since the study was cross-sectional, the temporal relationship between exposure and outcome variable could not be established. The study was conducted only in the rural part of the community. In addition, the study did not involve men in the quantitative data collection and the information about the husband which was indirectly obtained from women might have not represented them.

## Conclusion

The total demand for modern contraceptive methods in the study area was found to be high. Though contraceptive use was high, the uses of long-acting methods like IUCD and permanent methods were seen to very low. Woman’s age, number of children alive, couple’s intention for more children and discussions about contraceptive use among the couple were significantly associated with demand for modern contraceptives among women. Therefore, any program aimed at promoting family planning at the district level should look for ways and means of increasing demand for modern contraceptive methods in the study area. Service providers in the district should also provide necessary information regarding all methods of contraceptives to minimize fears that exist among women particularly on long-acting and permanent methods of family planning. Male involvement should be encouraged as couples’ discussion on family planning affected its utilization in the study area.

## Data Availability

Data will be available on request of the corresponding author.

## Abbreviations

CPR: Contraceptive Prevalence Rate
EDHS: Ethiopia Demographic and Health Survey
FGD: Focus Group Discussion
FP: Family Planning
HEWs: Health Extension Workers
IUCD: Intra Uterine Contraceptive Device
LAM: Lactational Amenorrhea Method
LAPM: Long-Acting and Permanent Methods
MDGs: Millennium Development Goals
MOH: Ministry of Health
MWRA: Married Women of Reproductive Age
NGOs: Non-Governmental Organizations
OCP: Oral Contraceptive Pills
SSA: Sub-Saharan Africa
TFR: Total Fertility Rate

## References

[1] IPPF. Understanding demand and supply for contraception. United Kingdom: Portfolio Publishing.com; 2011. p. 1–4.

[2] Kantorova ABV. Global trends in contraceptive method mix and implications for meeting the demand for family planning. New York; 2012.

[3] Abulie Takele GDMY (2012). Demand for long-acting and permanent methods of contraceptives and factors for non-use among married women of Goba Town, Bale Zone, South East Ethiopia: Madawalabu University.

[4] Madeleine S, Fabic YC, Bongaarts J, Darroch JE, Ross JA, Stover J (2014). Meeting demand for family planning within a generation: the post-2015 agenda.

[5] Ferede T (2013). Multilevel Modelling of Modern Contraceptive Use among Rural and Urban Population of Ethiopia. Am J Math Stat.

[6] Michael EJ (2012). Use of contraceptives methods among women in stable marital relations attending health facilities in Kahama District, Shinyanga region, Tanzania; Muhimbili University.

[7] Haile A (2009). Demand for long-acting and permanent contraceptive methods and associated factors among family planning service users.

[8] Gribble KRJ (2009). Expanding contraceptive choice: five promising innovations.

[9] Hatam Hosseini BB (2012). Demand for using contraceptive methods among Kurdish women in the city of Mahabad. Med Sci.

[10] Alem Gebremariam AA (2014). Intention to use long-acting and permanent contraceptive methods and factors affecting it among married women in Adigrat town, Tigray, Northern Ethiopia. Reprod Health.

[11] Prata Ndola (2009). Making family planning accessible in resource-poor settings. Philosophical Transactions of the Royal Society B: Biological Sciences.

[12] USAID, CfDaPAC, Partners in Population and Development, Africa Regional Office (PPD ARO), Population Reference Bureau (PRB) (2012). Family Planning in Ethiopia.

[13] Rahman M. A potential contraceptive method mix for the Ethiopian family planning program. Health. 2008:1–12.

[14] Central Statistical Agency [Ethiopia] and ICF International. Ethiopia Demographic and Health Survey 2011. Addis Ababa, Ethiopia and Calverton, Maryland, USA: Central Statistical Agency and ICF International; 2012.

[15] Worku WMA (2011). Determinants of low family planning use and high unmet need in Butajira District, south Central Ethiopia. Reprod Health.

[16] Doyore KCF (2014). Unmet Need for Family Planning and Associated Factors among Currently Married Women in Misha District. South Ethiop Public Health.

[17] Getiye Dejenu MAAAA (2013). Prevalence and associated factors of unmet need for family planning among married women in Enemay District, Northwest Ethiopia. Med Sci.

[18] Gizachew Abdissa Bulto TAZTKB (2014). Demand for long-acting and permanent contraceptive methods and associated factors among married women of the reproductive age group in Debre Markos town, North West Ethiopia. Women’s Health.

[19] Sarah EK, TNC B, Fishel JD, Westoff CF (2012). Revising Unmet Need for Family Planning.

